# Safety of fluralaner chewable tablets (Bravecto^TM^), a novel systemic antiparasitic drug, in dogs after oral administration

**DOI:** 10.1186/1756-3305-7-87

**Published:** 2014-03-07

**Authors:** Feli M Walther, Mark J Allan, Rainer KA Roepke, Martin C Nuernberger

**Affiliations:** 1 MSD Animal Health Innovation GmbH, Zur Propstei, Schwabenheim 55270, Germany

**Keywords:** Fluralaner, Dog, Safety, Bravecto™

## Abstract

**Background:**

Fluralaner is a novel systemic insecticide and acaricide that provides long acting efficacy in dogs after a single oral treatment. This study investigated the safety of oral administration of fluralaner in chewable tablets to dogs at the highest recommended treatment dose and at multiples of this dose.

**Methods:**

Thirty-two (16 male and 16 female) healthy 8-week old Beagle dogs weighing 2.0 - 3.6 kg at first administration were included in the study. Fluralaner was administered on three occasions at 8-week intervals at doses of up to 56, 168, and 280 mg fluralaner/kg body weight, equivalent to 1, 3, and 5 times the highest recommended treatment dose of fluralaner; sham dosed dogs served as controls.

During the study, all dogs were clinically observed, and their health was carefully monitored including body weight development, food consumption and measurement of hematology, coagulation, clinical chemistry (including measurement of levels of ACTH and C-reactive protein) and urinalysis. Following euthanasia of the dogs, complete gross post mortem examination, including organ weight determination, and histopathological examination of multiple tissues were conducted.

**Results:**

There were no clinical findings related to fluralaner treatment. Statistically significant differences between the treated groups and the control group were observed for some clinical pathology parameters and organ weights; none of these findings were considered to be of clinical relevance.

**Conclusions:**

Oral administration of fluralaner at the highest recommended treatment dose (56 mg/kg) at 8-week intervals is well tolerated and has a safety margin of more than five in healthy dogs eight weeks of age or older and weighing at least 2 kg.

## Background

Fluralaner is a novel systemically administered insecticidal and acaricidal product that provides long acting efficacy after oral administration to dogs. Fluralaner belongs to a new class of compounds with antiparasitic activity, the isoxazolines. These compounds have activity against γ-aminobutyric acid- (GABA-) and glutamate-gated chloride channels with significant selectivity for insect neurons over mammalian neurons [1]. A field study has shown that a single fluralaner dose administered orally to dogs provides at least twelve weeks of flea- and tick control [2]. This long duration of activity offers a more convenient treatment over monthly flea and tick control treatments with a potential compliance advantage.

This study was designed to demonstrate the safety of this systemic treatment and to investigate any possible health impact from repeated oral administration to healthy dogs of either the highest recommended dose or multiple overdoses.

## Methods

This randomized, parallel-group, blinded study included 32 (16 male and 16 female) healthy 8-week old Beagle dogs. A total of 24 dogs received fluralaner repeatedly and 8 sham dosed dogs served as controls. The study design was based on VICH GL 43 target animal safety requirements for veterinary pharmaceutical products [3].

This study was conducted in Ireland in compliance with the Irish national animal protection legislation framework. The study plan was approved by the ethics committee of Charles River Laboratories Preclinical Services Ireland Ltd. The experimental license number of the investigator was B100/3771.

Beagle pups were enrolled in the study on day -14 (start of acclimation) and were found to be healthy on initial physical and clinical pathological evaluation. Dogs received sulfadiazine and trimethoprim during acclimation for prophylaxis of coccidiosis, which might occur in dog colonies. Fecal samples collected from all dogs prior to study start revealed coccidia for several samples; however, all dogs were in good general health and therefore included in the study. Dogs were housed in a climate controlled room (16° - 20°C) with a day length of 10 hours light and 14 hours darkness and fed a standard commercial diet at recommended rates. Dogs were housed individually from day -3 to the end of the study. All dogs were vaccinated once against *Bordetella bronchiseptica* and canine parainfluenza virus and twice (5 weeks apart) against canine distemper virus, canine adenovirus 2, canine parvovirus, canine parainfluenza virus and *Leptospira interrogans* with the second vaccine given 22 days after the first fluralaner treatment/sham dosing. All dogs received praziquantel, pyrantel embonate, and febantel before and 22 days after the first fluralaner treatment/sham dosing for intestinal parasite infection. No clinical findings were observed in any dog in association with the vaccination or deworming.

Dogs were randomly allocated into groups using a block randomization procedure. Dogs were separated by gender and ranked in descending order of body weight. If two dogs had the same bodyweights, the dogs were sub-ranked by decreasing microchip number. The top four dogs within each gender formed a block that was randomly allocated to each of the four groups, and the process repeated until 4 male and 4 female dogs were allocated to each group. Three groups received fluralaner at different doses and one group served as sham dosed control.

The recommended dose range of fluralaner during routine clinical use is between 25 and 56 mg/kg [2,4]. This study evaluated the oral administration of fluralaner, formulated as a chewable tablet, at up to 1, 3, or 5 times the highest recommended treatment dose, i.e. up to 56 (1X group), 168 (3X group), or 280 (5X group) mg fluralaner/kg body weight. The tablet formulation used was the final commercial formulation intended to be marketed as Bravecto™ [MSD Animal Health, 4], produced under Good Manufacturing Practice (GMP). Dogs were administered fluralaner three times at 8-week (56 days) intervals on days 0, 56 and 112, with the first dose administered at 8 weeks (54–62 days) of age and 2.0 - 3.6 kg of body weight (Table 1). The dogs were weighed before each fluralaner treatment to calculate the appropriate dose. A single whole tablet or a combination of whole tablets, containing 112.5 mg or 250 mg fluralaner, were administered to each dog to deliver a dose as close as possible to the calculated dose (Table 2). Following tablet administration, a small amount of water was administered to encourage swallowing. Dogs from the control group were not administered fluralaner and were sham dosed with water on days 0, 56 and 112. On treatment days, dogs of all groups received a portion of their normal daily ration of food approximately 10 to 20 minutes prior to treatment and the remaining portion of the daily ration directly after treatment. Dogs were fed around the time of treatment to ensure high systemic fluralaner exposure, since fluralaner bioavailability is higher in fed dogs [5].

**Table 1:** Study dog body weights and ages at the time of first treatment

**Parameter**	**Group**				
		**Control**	**Fluralaner**	**Fluralaner**	**Fluralaner**
			**1X**	**3X**	**5X**
Body weight (kg)	Range	2.1 - 2.8	2.0 - 3.6	2.0 - 3.3	2.0 - 3.0
	Mean	2.6	2.8	2.8	2.7
Age (days)	Range	56 - 62	54 – 62	56 – 59	54 – 62
	Mean	58	57	57	58

**Table 2:** Fluralaner dose range administered to dogs in each group

**Treatment**	**Dose range per group (mg/kg)**		
	**Fluralaner**	**Fluralaner**	**Fluralaner**
	**1X**	**3X**	**5X**
First treatment (day 0)	31.3 – 56.3	139.4 – 168.8	255.2 – 281.3
Second treatment (day 56)	47.2 – 55.6	153.1 – 163.0	253.1 – 272.5
Third treatment (day 112)	43.1 – 55.3	151.5 – 166.0	254.2 – 277.8

Dogs were observed twice daily for general health throughout the study. In addition, all dogs were observed by a technician for signs like gagging, salivation, tablet regurgitation or vomiting during the first hour following each treatment. Detailed clinical assessments were performed by a veterinarian, who was blinded (masked) to the treatment each dog had received, before each treatment and at 1, 2, 3, 4, and 8 hours after each treatment. These examinations included assessments of abnormalities in behaviour, coat, locomotion, respiration, eyes (discharge, mydriasis, miosis, corneal opacity), mucous membranes, salivation, auscultation of heart, vomitus, feces and urine as present in pen, and any other visible abnormalities. Physical examinations were performed by a blinded veterinarian for all dogs on days -14, -7, -1, 1, 55, 57, 111, 113 and 167. These examinations included assessments of abnormalities in behaviour, locomotion, musculoskeletal system, coat, skin, superficial lymph nodes, eyes, pupils, ears, oral cavity, mucous membranes, capillary refill time, respiration, auscultation of heart and respiratory tract, heart rate, respiratory rate, pulse, abdominal palpation, rectal temperature, any other visible abnormalities and body condition scaled 1 (cachexia) to 5 (adiposity).

The individual food consumption was recorded daily and body weights were recorded weekly throughout the study. Blood samples were collected for clinical pathology (hematology, coagulation, clinical chemistry including ACTH and C-reactive protein; Table 3) before the first treatment and on days 8, 50, 106 and 162; and to monitor systemic exposure to fluralaner before and 2, 7, 14 and 28 days after each treatment. Urine samples were taken before the first treatment and on days 7/8, 49/50, 105/106 and 161/162.

**Table 3:** Clinical pathology parameters analyzed and mean results of day 162 for the control group and the fluralaner 5X group

	**Parameter (unit)**	**Control**	**fluralaner 5X**
**Hematology**	Basophils (x 10^9/L)	0.175	0.171
	Eosinophils (x 10^9/L)	0.368	0.748
	Hematocrit (L/L)	0.476	0.451
	Hemoglobin (g/dL)	15.24	14.29
	Large unclassified cells (x 10^9/L)	0.04	0.06
	Lymphocytes (x 10^9/L)	3.81	4.13
	Mean corpuscular hemoglobin (pg)	21.01	21.26
	Mean corpuscular hemoglobin concentration (g/dL)	31.99	31.65
	Mean corpuscular volume (fL)	65.69	67.14
	Monocytes (x 10^9/L)	0.56	0.60
	Neutrophils (x 10^9/L)	6.67	6.65
	Platelets (x 10^9/L)	506	536
	Red blood cells (x 10^12/L)	7.24	6.74
	Reticulocytes (x 10^9/L)	70.8	53.3
	Total white blood cells (x 10^9/L)	11.62	12.34
**Coagulation**	Activated partial thromboplastin time (s)	12.78	13.59
	Fibrinogen (mg/dL)	128.3	172.0
	Prothrombin time (s)	6.08	6.16
**Clinical**	Adrenocorticotrophic hormone (pg/mL)	32.89	34.68
**chemistry**	Alanine aminotransferase (U/L)	41.9	39.3
	Albumin (g/L)	34.75	33.13
	Albumin/globulin ratio	1.96	1.65
	Alkaline phosphatase (U/L)	70.50	72.00
	Amylase (U/L)	900	800
	Aspartate aminotransferase (U/L)	37.63	35.75
	Calcium (mmol/L)	2.77	2.76
	Chloride (mmol/L)	113.50	115.13
	Cholesterol (mmol/L)	5.96	5.68
	Creatine kinase (U/L)	306.5	223.4
	Creatinine (μmol/L)	54.25	50.50
	C-reactive protein (μg/mL)	3.84	10.16
	Gamma glutamyl transpeptidase (U/L)	3.50	3.13
	Globulin (g/L)	18.13	20.38
	Glucose (mmol/L)	6.24	6.24
	Inorganic phosphate (mmol/L)	2.14	2.14
	Lactate dehydrogenase (U/L)	32.50	23.88
	Magnesium (mmol/L)	0.69	0.71
	Phospholipid (mmol/L)	6.03	5.83
	Potassium (mmol/L)	4.75	4.75
	Sodium (mmol/L)	148.6	147.8
	Total bile acids (μmol/L)	5.98	4.51
	Total bilirubin (μmol/L)	1.78	2.10
	Total protein (g/L)	52.88	53.38
	Triglycerides (mmol/L)	0.44	0.34
	Urea (mmol/L)	5.20	4.08
**Urinalysis**	Bilirubin (mg/dL), ketones (mg/dL), glucose (mg/dL), turbidity	0	0
	Blood pigments (mg/dL)	0.029	0.004
	Leukocytes (cells/μL)	150	106
	pH	6.44	6.44
	Protein (mg/dL)	8.75	5.00
	Specific gravity	1.031	1.025
	Urobilinogen (mg/dL)	0.40	0.20
	Volume (mL)	133	153
	Urine color	Light yellow - yellow	
	Microscopic examination (crystals, casts, red blood cells, white blood cells, epithelial cells, bacteria, other abnormalities)	Similar frequency distribution	

To complete the safety assessment, all dogs underwent a post mortem examination, as required by VICH GL 43 [3]. On day 168, all dogs were sedated by intramuscular injection of ketamine and xylazine and thereafter euthanized by intravenous injection of sodium pentobarbitone as per study plan. A complete post-mortem examination was performed on all dogs under the supervision of a blinded veterinary pathologist. Selected organs were weighed and multiple tissues were examined histopathologically (Table 4). Tissue samples were formalin-fixed (epididymides and eyes were fixed in Davidson’s fixative, and testes in modified Davidson’s fixative) and paraffin-embedded. Microscopy slides were stained with hematoxylin and eosin stain. Additional formalin-fixed frozen oil red O stained slides were prepared for heart, kidney and liver to ascertain the presence of fat. Femur bone marrow smears were prepared and stained with May Grunewald’s Giemsa stain. All samples were assessed by a veterinary histopathologist.

**Table 4:** Table of organs and tissues examined histopathologically and of organs weighed

**Organ/tissue examined histopathologically**	**Organ weighed**
Brain	√
Heart	√
Liver	√
Spleen	√
Kidneys	√
Pituitary gland	√
Thymus gland	√
Thyroid and parathyroid glands	√
Adrenal glands	√
Testes	√
Epididymides	√
Prostate gland	√
Ovaries	√
Uterus with cervix	√
Trachea, lung	-
Eyes, sciatic nerve	-
Different sections of spinal cord	-
Submandibular salivary gland	-
Tongue, oesophagus	-
Larynx, pharynx	-
Aortic arch	-
Gall bladder	-
Pancreas	-
Different section of gastro-intestinal tract, Peyer’s patches	-
Femur, stifle joint with bone, sternum	-
Skeletal muscle	-
Skin, mammary gland	-
Lymph nodes (submandibular, bronchial, mesenteric)	-
Urinary bladder	-
Vagina	-
Gross lesions	-

Body weight, food consumption (averaged over weekly intervals), physical examination results, clinical pathology parameters and absolute and relative organ weights were statistically compared between groups (SAS^®^ Language: Reference, Version 9.3, SAS Institute Inc., Cary, NC, USA) using 2 sided tests with the individual dogs as the experimental unit to evaluate the hypothesis that there are no differences between the groups. Body weight, food consumption, clinical pathology parameters (including numeric urinalysis parameters), heart rate and rectal temperature were analyzed using a repeated measured mixed model analysis of covariance. Categorical urinalysis parameters were analyzed using descriptive statistics. Organ weights were analyzed using analysis of variance models. Frequency distributions of the numbers of animals with abnormalities during clinical assessments or physical examinations were compiled for categorical parameters.

For clinical pathology parameters study-specific reference ranges were compiled, as these values were considered most suitable for the dog population examined. These reference ranges included results from the control group at all collection time points (before the first sham dosing and on days 8, 50, 106 and 162) and from the fluralaner-treated groups before the first treatment. In support, reference ranges from historical controls, the clinical pathology laboratory or from literature may be used to assess results [6]. All clinical pathology parameters found to be statistically significantly different were compared with these study-specific reference ranges to evaluate the clinical relevance. Individual values outside these reference ranges were assessed for possible clinical relevance. Clinical relevance was assessed by the veterinary investigator based on the following criteria: transience (temporary observation), dose–response relationship, values close to or within the reference ranges, association with evidence of clinical signs and with tissue changes on gross post mortem or histopathological examination.

The veterinary investigator assessed all parameters recorded and any findings for their relationship to fluralaner treatment. Any clinically relevant treatment-related findings were classified as adverse events.

## Results and discussion

There were no findings related to the treatment with fluralaner for any of the parameters assessed.

The 8-week treatment interval used in the present study is expected to be shorter than the recommended 12-week treatment interval for fluralaner tablets in clinical veterinary practice [2,4]. An 8-week treatment interval was selected in this study to provide data for clinicians who may select a shorter treatment interval under field conditions [4], and to provide an additional safety margin compared with the expected field use conditions.

The highest fluralaner dose administered during the study was in an eight-week old dog in the 5X group (281.3 mg/kg; Table 2). Fluralaner was administered on three occasions as previous pharmacokinetic data (unpublished observations) have shown that three treatments are sufficient to achieve steady state plasma concentrations. Administration of three sequential treatments at 8-week intervals produced a 24-week study period with the highest systemic exposure to fluralaner at doses of five times the highest recommended treatment dose. To further ensure peak plasma concentrations in treated dogs, fluralaner was administered at the time of feeding, since fluralaner bioavailability is higher in fed dogs [5]. All dogs partially or totally consumed the diet offered prior to the first treatment (day 0) and totally consumed the diet offered prior to the second and third treatment (day 56 and 112).

Fluralaner was quantifiable in the plasma of all treated dogs, confirming absorption and systemic exposure.

Throughout the 24-week study, no differences in growth rates or food consumption between the treated groups and the control group were observed.

All dogs were carefully observed for any clinical findings during the first hours following treatment to cover the period of rapidly increasing systemic fluralaner exposure [7] when acute clinical signs would most likely be apparent. However, no clinical findings related to the treatment with fluralaner were observed throughout these frequent observations following any of the fluralaner treatments on days 0, 56 and 112, or throughout the remainder of the study. Two cases of vomiting occurred during the study. One dog treated with a 1X dose for the first time vomited 4 hours after treatment. This dog was not administered a second dose based on the canine gastric emptying half-time of approximately 3 hours in fed dogs [8]. Absorption of the administered dose was confirmed by dose-related plasma concentrations of fluralaner. This dog remained in the study and did not show any other clinical signs (including vomiting) in the remainder of the study. Another dog developed signs of gastroenteritis (vomiting and diarrhea) 5 days after administration of the first fluralaner dose at 3 times the highest recommended treatment dose. The dog was treated with injectable antibiotics (enrofloxacin) and clinical signs resolved fully over the next four days. These clinical signs were considered to be unrelated to fluralaner administration due to the 5-day interval between fluralaner treatment and the onset of clinical signs, as well as the subsequent rapid resolution of clinical signs following antibacterial treatment. This dog did not show any further signs of gastroenteritis and remained in the study.

Occasional clinical findings were observed in individual dogs from the treated and control groups, during the 168-day study (Table 5). Clinical findings included incidences of mild abnormal feces (small amount of loose feces, small amount of mucoid feces/mucus in feces, traces of fresh blood), slight reductions in body condition score (2 or 2–3 on a scale of 1–5), or mild superficial skin injury; all of these were minor and none affected the general health condition of dogs. These observations were considered to be unrelated to fluralaner treatment because they occurred at a similar or lower incidence in the treated groups compared with the control group and without a dose–response relationship. In addition, abnormal feces were observed before the first treatment across all groups. Although all dogs were treated with endoparasiticides, intestinal parasite infections, like coccidia or giardia, may have been supportive in the occurrence of incidences of mild abnormal feces observed pre- and post-treatment. Based on the frequency distribution of the number of dogs with abnormal findings during clinical assessments or physical examinations, there were no dose-related differences between groups (Table 6). Treated dogs were found to have statistically significant lower mean rectal temperatures than control dogs at six time points (days 55 and 167 – 1X group; days 55, 111 and 167 – 3X group; and day 55 – 5X group). This difference was not considered to be of clinical relevance because there were no clinical signs or significant changes in clinical pathology in the treated dogs, and the rectal temperatures of all treated dogs were within the range observed in control dogs.

**Table 5:** Clinical findings in dogs of treated and control groups following the first treatment

**Observation**	**Number of dogs affected**	**Analysis**	**Conclusion**
Scattered incidences of abnormal feces (loose, mucoid, traces of fresh blood)	Total observed: n = 15	Similar number of animals affected in each treated group compared to controls; abnormal feces already observed pre-treatment across all groups	Not treatment related, no adverse event
	Control group: n = 4		
	1X group: n = 5		
	3X group: n = 3		
	5X group: n = 3		
Reduced body condition score (2 or 2–3 on a scale of 1–5)	Total observed: n = 8	Similar (3X group, 5X group) or lower (1X group) number of animals affected compared to controls; no dose–response relationship	Not treatment related, no adverse event
	Control group: n = 3		
	1X group: n = 0		
	3X group: n = 3		
	5X group: n = 2		
Localized hair thinning/pressure wound/wound	Total observed: n = 6	Similar (1X group, 5X group) or lower (3X group) number of animals affected compared to controls; no dose–response relationship	Not treatment related, no adverse event
	Control group: n = 2		
	1X group: n = 3		
	3X group: n = 0		
	5X group: n = 1		
Vomiting of food after first treatment, no other clinical findings	Total observed: n = 1	Single case; no dose–response relationship; not observed following repeated treatment	Not treatment related, no adverse event
	Control group: n = 0		
	1X group: n = 1		
	3X group: n = 0		
	5X group: n = 0		
Gastroenteritis with inappetence, dullness, vomit	Total observed: n = 1	Single case; no dose–response relationship; not observed following repeated treatment	Not treatment related, no adverse event
	Control group: n = 0		
	1X group: n = 0		
	3X group: n = 1		
	5X group: n = 0		

**Table 6:** Frequency distribution by group of dogs with abnormalities recorded following the first treatment

**Time point**	**No. of animals with abnormalities recorded per group**				**Analysis**	**Conclusion**
	**Control group**	**1X group**	**3X group**	**5X group**		
**Clinical assessment**	2	5	1	1	Number of animals with abnormalities higher in 1X group than in controls, but similar to controls in 3X and 5X group; no dose–response relationship	Not treatment related
**Physical examination**	4	4	4	3	Number of animals with abnormalities similar to controls; no dose–response relationship	Not treatment related

Statistically significant differences between the treated groups and the control group were observed for some clinical pathology parameters and organ weights and a few individual single time point clinical pathology results fell outside the reference ranges. All of these findings were evaluated based on the assessment criteria and were not considered to be clinically relevant. Gross post mortem and histopathological examinations found no notable differences between the treated groups and the control group (data not shown).

The evaluation of the present study is consistent with the conclusion drawn by authorities [4].

## Conclusions

This detailed evaluation of the safety of fluralaner, a novel systemic antiparasitic drug, following oral administration at doses much higher than the recommended treatment dose at 8-week intervals, did not reveal any adverse events.

Oral administration of fluralaner, formulated as a chewable tablet, to healthy dogs at dose rates of up to 281.3 mg/kg on three occasions at 8-week intervals did not lead to any treatment-related findings that could be detected through careful clinical observation, clinical pathological evaluation or on gross or microscopic post mortem examination. Oral administration of fluralaner at the highest recommended treatment dose (56 mg/kg) is well tolerated by dogs and has a safety margin of more than five in healthy dogs eight weeks of age or older and weighing at least 2 kg.

## Competing interests

FMW, MJA, RKAR and MCN are employees of Merck / MSD Animal Health.

## Authors’ contributions

FMW, MJA, RKAR and MCN authored the study design, monitored the study and interpreted the results. All authors revised and approved the final version of the manuscript.
